# Editorial: Epidemiology of orthopaedic sports trauma and injuries

**DOI:** 10.3389/fspor.2026.1818539

**Published:** 2026-03-30

**Authors:** Amir Human Hoveidaei, Jack Parker, Mohammad Razi, Ramona Ritzmann, Jakob Adolf, Sean A. Tabaie

**Affiliations:** 1 International Center for Limb Lengthening, Rubin Institute for Advanced Orthopedics, Sinai Hospital of Baltimore, Baltimore, MD, United States; 2 Department of Orthopaedic Surgery, Rasoul Akram Hospital, University of Medical Sciences, Tehran, Iran; 3 Department of Sport and Sport Science, University of Freiburg, Freiburg, Germany; 4 Innovation Translation Center, AO Foundation, Davos, Switzerland; 5 Department of Paediatric Orthopaedics, Sofia Medical University, Sofia, Bulgaria; 6 Department of Orthopedic Surgery, Nationwide Children’s Hospital, Columbus, OH, United States

**Keywords:** athletic injuries, epidemiology, incidence, injury prevention, musculoskeletal injuries, risk factors, sports, sports epidemiology

Every weekend, millions of young athletes take the field, court, or ice, and a substantial number will experience injuries that could potentially have been prevented. Sports-related trauma generates approximately 2.6 million emergency room visits annually among youth and young adults in the United States, with direct medical costs approaching $2 billion per year (1). These figures reflect the considerable personal and societal impact of orthopaedic trauma, from lost playing time to long-term health consequences. Even a single injury type, such as anterior cruciate ligament tears, costs the US economy an estimated $3.6 billion annually, while in some countries individual sports can generate in excess of $100 million in injury-related costs per year (2). Importantly, a growing body of evidence suggests that many of these injuries are preventable (3). The same epidemiologic research that identifies higher injury rates in certain sports, age groups, and settings also reveals specific opportunities for intervention (3, 4). By systematically tracking which sports injure the most athletes, which body regions are most vulnerable, and which mechanisms lead to harm, clinicians and researchers can implement evidence-based prevention programs that have been shown to meaningfully reduce injury rates (2, 5). Understanding the epidemiology of orthopaedic sports trauma is therefore not simply an academic exercise, but a practical roadmap for keeping athletes healthy, active, and safe.

Epidemiology, the study of the distribution and determinants of health-related events in populations, provides the foundation for modern clinical decision-making. Through systematic observation, epidemiology allows clinicians to move beyond individual experience to recognize patterns of injury and disease, identify modifiable risk factors, and evaluate the effectiveness of interventions. With roots in infectious disease (6), its methods are now central to every area of medicine, including the prevention and treatment of musculoskeletal injuries.

In orthopaedics, where trauma and sports-related injuries account for a substantial proportion of healthcare utilization, epidemiologic research is particularly valuable. Understanding who becomes injured, how injuries occur, and under what circumstances they arise is essential for guiding prevention strategies, optimizing systems of care, and improving outcomes. The present Research Topic brings together studies that examine the epidemiology of injuries across a range of popular sports, many of which carry significant injury risk.

A central goal of sports injury epidemiology is prevention. Liu et al. addressed this goal directly through a meta-analysis of 16 randomized controlled trials evaluating multicomponent injury-prevention programs in adolescent team athletes. Their findings highlight the importance of balance training, lower-extremity and core strengthening, and structured warm-up routines, interventions that are practical and easily implemented across youth sports. These results reinforce the concept that well-designed prevention programs can meaningfully reduce injury risk at a population level (Liu et al.).

Advances in methodology continue to expand the tools available to sports injury researchers. West et al. demonstrated this progress through a study of field hockey injuries using detailed video-based coding to analyze the game situations surrounding injuries. By examining injuries in relation to specific match contexts and standardizing results to 10-minute segments of game play, the authors were able to move beyond simple injury counts and provide insight into the circumstances that place athletes at risk. Their comparison of outdoor, indoor, and Hockey5s formats showed no significant differences in injury rates across game types or between genders, illustrating how modern analytic approaches can inform rule changes, coaching strategies, and player safety (West et al.).

Beurienne et al. applied a similarly practical epidemiologic approach to the increasingly popular sport of bouldering. Using an online survey of climbers, the authors characterized behaviors associated with falls, the mechanisms by which said falls occurred, and the specific injuries that resulted. By identifying common scenarios that lead to injury, their study offers actionable information for athletes and coaches. This work exemplifies the value of descriptive epidemiology, in which documentation of real-world patterns that can guide prevention efforts (Beurienne et al.).

Other contributions in this Research Topic address sports that have historically received limited attention in the epidemiologic literature. Gondouin et al. provided one of the most comprehensive recent analyses of injuries among fencers. Their findings revealed that the lower extremity is the most commonly affected area and that injury patterns meaningfully vary according to the weapon used. These sport-specific insights are essential for tailoring conditioning programs and protective strategies to the unique demands of fencing (Gondouin et al.). Rincón et al. performed a systematic review of injuries in artistic swimming, another discipline with sparse data. The authors highlighted inconsistencies in surveillance methods and demonstrated that many studies fail to adhere to recommended standards for injury reporting. Despite these limitations, they were able to summarize available evidence regarding injury incidence, location, and type, providing an important foundation for future research. Their work underscores the ongoing need for standardized data Research Topic methods in order to generate reliable epidemiologic information (Rincón et al.).

Even in well-established sports, new perspectives can yield valuable insights. Prioul et al. examined American football from an international vantage point, analyzing playing styles and injury patterns among U19 European championship teams. By comparing Balanced, Running, and Passing styles of play, the authors showed that different approaches influence injuries, offering practical implications for coaching strategies and workload management. This study demonstrates how epidemiology can be applied not only to identify risks but also to understand how variations in competitive approach affect athlete safety (Prioul et al.).

Although these studies examine different sports and populations, several common themes emerge. First, many sports, including fencing and artistic swimming, remain underrepresented in the epidemiologic literature, emphasizing the need for broader surveillance efforts. Second, methodologic innovation, such as video-based injury coding and online behavioral surveys, is expanding the tools available to researchers. Finally, across all studies, the ultimate objective is the same: translating descriptive data into practical strategies that reduce injury risk.

Collectively, the studies presented in this Research Topic illustrate the breadth and practical importance of sports injury epidemiology. Whether through meta-analyses of prevention programs, innovative methods of injury surveillance, or the characterization of previously understudied athletic populations, each contribution adds to the evidence base to keep athletes safe. Continued investment in high-quality epidemiologic research will remain essential for informing coaches, clinicians, and policymakers, and ultimately for preventing injuries before they occur.
